# Supplementary material for: Platelet Inhibition by Low-Dose Acetylsalicylic Acid Reduces Neuroinflammation in an Animal Model of Multiple Sclerosis
Source: Int J Mol Sci. 2021 Sep 14;22(18):9915. doi: 10.3390/ijms22189915 (PMC8465626; doi:10.3390/ijms22189915)
Supplement: Supplementary file 1 [file ijms-22-09915-s001.zip › ijms-1356189-supplementary.pdf]

International Journal of
Molecular Sciences

MDPI

Supplemental figures

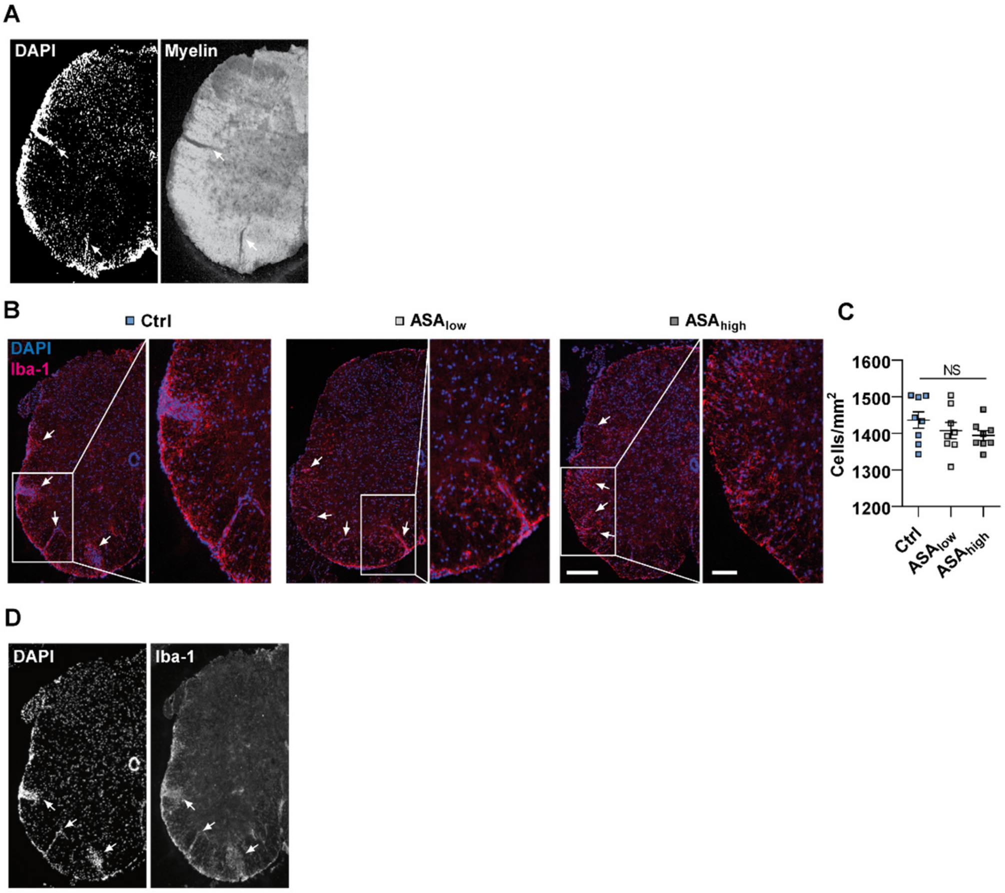

Figure S1. *Treatment with acetylsalicylic acid (ASA) has no effect on microglia or macrophage activation. (A) Single stainings of nuclei (DAPI) and myelin (FlouroMyelin) of lumbar spinal cord sections obtained from EAE mice isolated at disease maximum are shown. (B) Aforesaid spinal cord sections of all experimental groups (Ctrl, ASA$_{low}$, ASA$_{high}$) were stained for ionized calcium-binding adaptor molecule 1 (Iba-1). Representative images are shown (scale bar: 200 µm; close-up scale bar: 100 µm) and arrows indicate regions with a high density of Iba-1$^+$ cells. (C) The number of Iba-1$^+$ cells per mm^2 lesion was calculated and the quantitative analysis is shown. (D) Single stainings of DAPI and Iba-1 of spinal cords obtained from EAE mice are presented. Data were analyzed by Mann-Whitney U-test and each symbol represents an individual mouse (8 vs. 8 vs. 8 mice; 2 independent experiments; C). The level of significance was labeled as NS (not significant).*

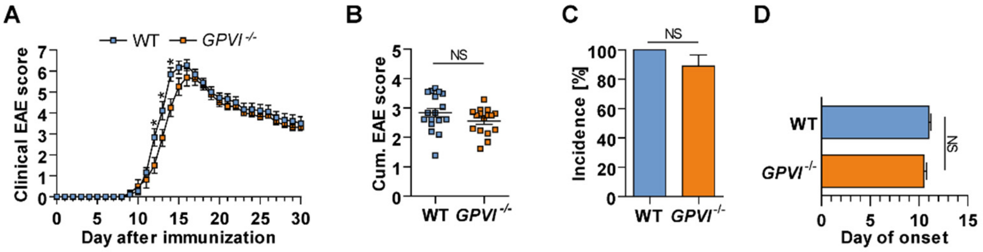

Figure S2. *Glycoprotein VI deficiency merely has a marginal impact on the clinical outcome of EAE. (A, B) Daily clinical EAE scores of $MOG_{35–55}$-immunized mice are shown (A) and the cum. EAE scores are presented for WT and GPVI-deficient ($GPVI^{-/-}$) mice (B). (C, D) Disease incidence (C) and the mean day of disease onset after immunization (D) are given. Data were analyzed by two-way ANOVA (n = 18 vs. 18 mice; three independent experiments; A) or Mann-Whitney U-test (B, C, D). Each symbol represents an individual mouse (B). The level of significance was labeled as NS (not significant).*
